# Scrub Typhus With an Unknown Source of Infection

**DOI:** 10.7759/cureus.90324

**Published:** 2025-08-17

**Authors:** Takeyoshi Honta, Mami Kida, Seiji Chubachi

**Affiliations:** 1 Department of Internal Medicine, Kurihara Central Hospital, Kurihara, JPN; 2 Department of General Surgery, Kurihara Central Hospital, Kurihara, JPN

**Keywords:** infection, orientia tsutsugamushi, scrub typhus case report, tick, tsutsugamushi

## Abstract

Scrub typhus, caused by *Orientia tsutsugamushi*, is a mite-borne zoonotic disease endemic to many parts of Asia. Although traditionally associated with rural environments, cases of urban transmission are increasingly being reported, possibly due to ecological changes such as urban heat islands, which may expand the habitats of vector mites. Recognizing these emerging patterns is important, as scrub typhus often presents with non-specific clinical features, making diagnosis challenging and potentially delaying appropriate treatment, thereby worsening patient outcomes. We report a case of an 84-year-old woman who presented with a seven-day history of anorexia, malaise, and headache. Physical examination revealed mild altered consciousness, an eschar on the left eyelid, and a maculopapular rash on the trunk. Laboratory findings included thrombocytopenia, elevated liver enzymes, and increased C-reactive protein (CRP). Despite no known exposure to grasslands or brush fields, and with no recent travel, animal contact, or other potential exposure routes identified, scrub typhus was suspected based on clinical findings. She was treated with intravenous minocycline for 14 days, following which the fever resolved and laboratory abnormalities improved. The antibody test for *Orientia tsutsugamushi* was positive. Her clinical course was favorable, and she was discharged in good condition. Although scrub typhus is traditionally associated with rural environments, urban transmission is becoming more recognized, possibly due to ecological changes such as urban heat islands, where temperatures are higher than in surrounding rural areas, potentially expanding the habitats of vector mites. This case highlights that scrub typhus should be considered in patients presenting with fever, rash, and eschar, even when no clear environmental exposure is identified.

## Introduction

Scrub typhus, caused by *Orientia tsutsugamushi*, is a rickettsial zoonotic disease transmitted by the bite of infected chigger mites [1,2]. It is endemic in many parts of Asia, particularly in Japan, Taiwan, and South and Southeast Asian countries such as India, Myanmar, Sri Lanka, and Pakistan, and remains a significant public health concern in many rural areas [3]. Transmission typically occurs in environments such as mountainous regions, grasslands, and riverbanks, where the vector mites are commonly found [1].

Clinically, scrub typhus is characterized by a triad of fever, rash, and eschar at the site of the mite bite [2]. In addition to these hallmark features, patients often exhibit laboratory abnormalities such as elevated liver enzymes and thrombocytopenia [2]. While tetracyclines are generally effective in treating the disease, diagnosis can be challenging due to the non-specific nature of the initial febrile presentation, which may resemble other common conditions such as influenza, viral upper respiratory infections, or other bacterial infections [2,4]. In addition, although traditionally associated with rural environments, urban and peri-urban transmission has also been increasingly reported in recent years [5]. Therefore, this challenge is even greater in atypical exposure scenarios, where the absence of a rural exposure history may delay clinical suspicion.

Delayed diagnosis and treatment may lead to severe complications, including disseminated intravascular coagulation (DIC), multi-organ failure, and death [2]. Therefore, early recognition of the disease and prompt antibiotic therapy are essential for improving patient outcomes. Here, we report a rare case of scrub typhus presenting with an eschar on the eyelid in a patient without any rural exposure history. While scrub typhus remains a significant cause of febrile illness across Asia, its occurrence in our locality underscores the need for heightened clinical awareness and public health surveillance, even in urban and peri-urban settings.

## Case presentation

**Figure 1:**
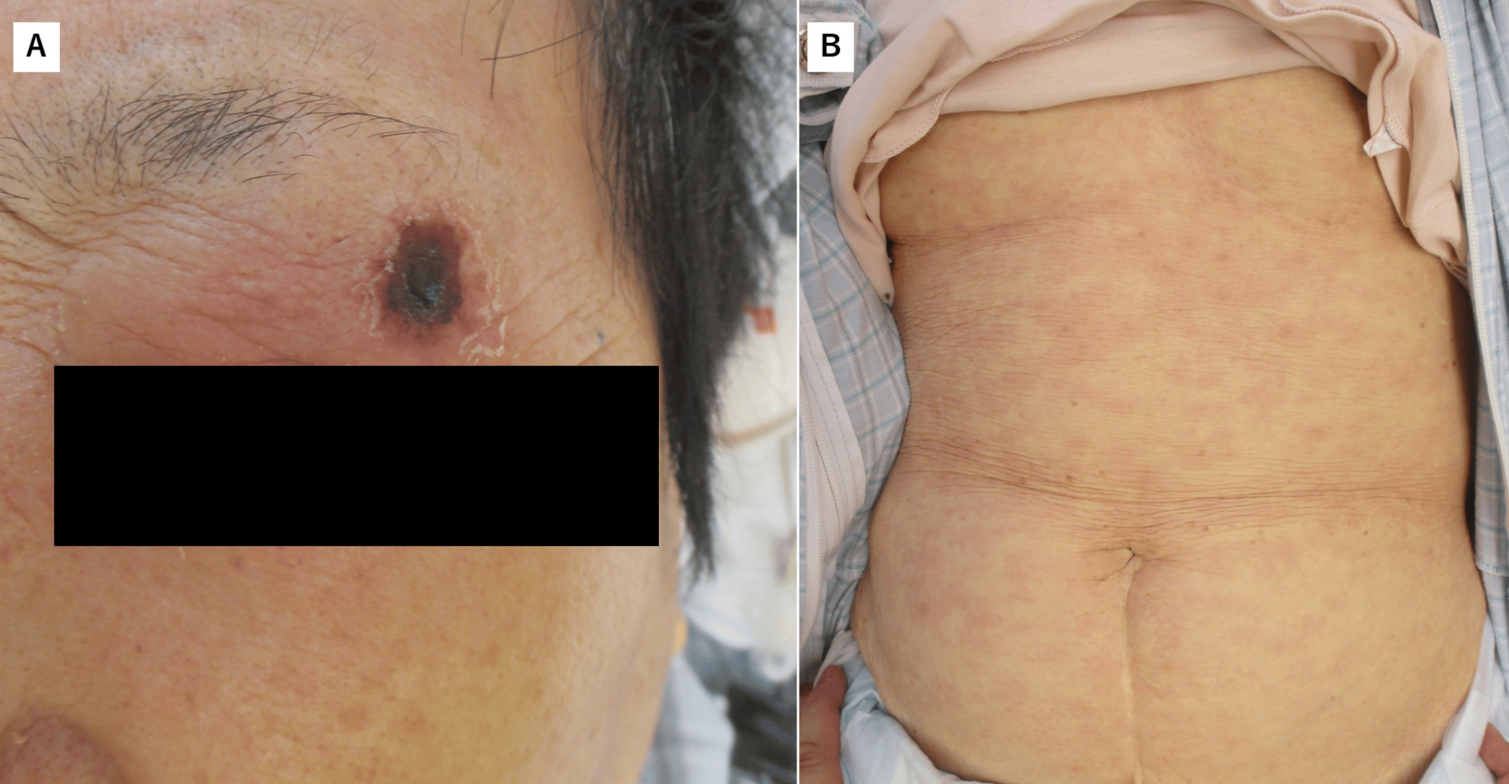
An eschar and a rash These images show an eschar on the left upper eyelid, approximately 10 mm in diameter (A), and a maculopapular rash on the trunk (B). Written informed consent to include the images in an open-access article was obtained from the patient.

An 84-year-old woman was referred to our department because of loss of appetite, malaise, and headache for seven days. There was no significant past medical history or family history. The patient had no history of exposure to brush fields or grasslands, and no recent travel, animal contact, or other potential exposure routes such as gardening or visiting parks. Physical examination revealed mild consciousness disorder, an eschar on the left eyelid (Figure 1A), and a maculopapular rash on the trunk (Figure 1B). Laboratory tests revealed a low platelet count (130,000/μL), hepatic dysfunction (aspartate aminotransferase, 57 U/L; alanine aminotransferase, 26 U/L), and an elevated C-reactive protein (CRP) level (8.25 mg/dL). After admission, the patient developed a fever of 40 °C.

The differential diagnosis included viral infections such as influenza or COVID-19, as well as allergic disorders. However, because of the characteristic eschar and the fact that scrub typhus is relatively common in our hospital’s service area, tsutsugamushi disease was strongly suspected.

**Figure 2:**
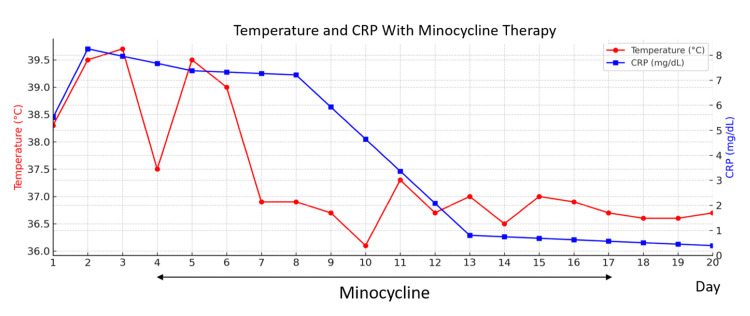
Clinical course of temperature and CRP following minocycline therapy Minocycline (200 mg/day) was administered intravenously for 14 days, from day 4 to day 17. The patient's fever persisted above 39°C until day 8 but rapidly resolved thereafter. CRP levels also decreased in parallel. Day numbers indicate the number of days from hospital admission. CRP: C-reactive protein

Therefore, an *Orientia tsutsugamushi* antibody test for IgG and IgM antibodies against the Gilliam, Kato, and Karp strains was performed using an indirect immunofluorescence assay (cut-off value ≥1:10 for each strain and antibody class) (Table 1), and intravenous minocycline (200 mg daily) was initiated on day 4 and continued for 14 days. Tests for the influenza virus and SARS-CoV-2 were also conducted and came out negative. After initiation of minocycline, the fever resolved and CRP levels normalized by day 10 (Figure 2), and platelet count and liver function returned to normal by day 19 (Table 1). Antibody tests for the Gilliam, Kato, and Karp strains (IgG and IgM) were positive on two separate occasions during hospitalization (Table 1), confirming the diagnosis. The patient's clinical course remained favorable, and she was discharged in good condition without any complications. In this case, the resolution of fever was accompanied by a marked decrease in CRP levels, a laboratory trend consistent with that reported in typical scrub typhus cases [2].

**Table 1:** Laboratory parameters and antibodies Laboratory findings from day 1 to day 19 are shown. Intravenous minocycline (200 mg/day) was initiated on day 4. On the same day, both IgG and IgM antibodies for *Orientia tsutsugamushi *(Gilliam, Kato, and Karp strains) were detected, with further elevation by day 19. CRP, WBC, and neutrophil counts gradually decreased following treatment, indicating clinical improvement. WBC: white blood cells; Hb: hemoglobin; Neutro: neutrophils; Plt: platelets; AST: aspartate transaminase; ALT: alanine transaminase; LD: lactate dehydrogenase; ALP: alkaline phosphatase; T-bil: total bilirubin; CRP: C-reactive protein; IgM: immunoglobulin M; IgG: immunoglobulin G

Parameter	Day 1	Day 3	Day 4	Day 7	Day 12	Day 19	Reference
WBC (10²/μL)	43.9	68	-	79.1	60	39.3	33-86
Hb (g/dL)	12	11.5	-	11.1	11.5	11.1	11.6-14.8
Neutro (%)	78.2	89	-	62	46.8	43.8	42.4-75.0
Plt (10⁴/μL)	13	17.4	-	17.4	22.7	18.3	15.8-34.8
AST (U/L)	57	65	-	66	28	23	13-30
ALT (U/L)	26	45	-	57	39	17	7-23
LD (U/L)	294	366	-	409	262	197	124-222
ALP (U/L)	101	102	-	130	116	106	38-113
T-bil (mg/dL)	0.6	0.5	-	0.5	0.9	1	0.4-1.5
CRP (mg/dL)	8.25	7.38	-	7.21	0.8	0.39	0.00-0.14
IgG Gilliam	-	-	160	-	-	1280	0-10
IgG Kato	-	-	160	-	-	1280	0-10
IgG Karp	-	-	10	-	-	320	0-10
IgM Gilliam	-	-	80	-	-	1280	0-10
IgM Kato	-	-	80	-	-	1280	0-10
IgM Karp	-	-	10	-	-	80	0-10

## Discussion

Scrub typhus is typically transmitted by the bite of chigger mites, which are usually found in grassy fields, bushes, and forested environments [1]. The classical clinical features include fever, rash, eschar at the site of the mite bite, thrombocytopenia, and hepatic dysfunction [2]. The presence of an eschar at the site of the bite is a hallmark finding and can be highly suggestive of the disease. However, eschars are not always easily detectable [4]. The challenge in diagnosis can result in treatment delays, potentially worsening patient outcomes.

In this case, the patient had no apparent history of outdoor activity or exposure to typical high-risk environments such as fields or forests where vector mites are commonly found. Despite the absence of an identifiable source of infection, the diagnosis of scrub typhus was made based on the combination of clinical signs and laboratory data.

Although scrub typhus has traditionally been associated with rural environments, recent studies have reported cases occurring in residential and peri-urban areas [5,6]. Park et al. reported scrub typhus cases in Seoul, South Korea, occurring in urban settings, including city parks and residential areas [6]. These findings suggest that transmission is not limited to conventional rural routes. In addition, Lou et al. suggested that urban heat islands and ecological changes may contribute to the expansion of suitable habitats for chigger mites [5].

Moreover, molecular epidemiological studies have highlighted the genotypic diversity of *Orientia tsutsugamushi *strains isolated from different geographical and ecological settings [7,8]. In particular, certain urban or peri-urban cases have been associated with atypical or previously uncharacterized genotypes, distinct from the classical rural strains such as Gilliam, Karp, and Kato [7,8]. This genetic variation suggests that some strains of *Orientia tsutsugamushi *may have developed characteristics that help them survive in new environments, including urban areas. These adaptations could include changes in how the bacteria interact with their mite or animal hosts and/or increased resistance to environmental conditions. Although molecular typing was not performed in this case, the possibility of urban transmission or strain diversity remains speculative. Further research combining longitudinal urban vector surveillance with genomic analyses, such as whole-genome sequencing, is needed to determine whether the appearance of scrub typhus in cities is due to the spread of existing strains into urban areas or the emergence of novel strains specifically adapted to urban environments.

Our hospital has confirmed seven cases of tsutsugamushi disease over the past 10 years, indicating that our region is one of the areas in Japan with a relatively high number of reported cases [9]. Although scrub typhus has long been associated with rural environments, the possibility of urban transmission must be considered, particularly when patients reside in endemic areas. In this case, the absence of a typical exposure route raises the suspicion of an alternative mode of transmission, possibly in an urban setting. Therefore, in endemic regions, it is essential to strengthen epidemiological surveillance and vector control strategies, not only targeting traditional rural areas but also expanding monitoring efforts to include urban environments, where undetected transmission may occur.

Early diagnosis and timely initiation of treatment with tetracyclines remain critical to preventing severe complications such as DIC, multi-organ failure, and even death [2]. However, diagnosis can be particularly challenging in cases without a clear exposure history or in patients infected in non-traditional settings. This highlights the importance of clinician awareness, especially in endemic regions, where it is essential to focus on clinical signs such as fever, rash, and eschar, and not depend on a history of exposure to high-risk environments. In addition, recognizing that scrub typhus can occur even in urban environments is essential for both clinical practice and public health efforts. Clinicians should stay alert to the possibility of urban transmission, and public health authorities may need to consider enhanced surveillance and vector control measures beyond traditional rural settings.

## Conclusions

This patient had tsutsugamushi disease with an unknown route of infection, but was diagnosed based on clinical symptoms and successfully treated. The diagnosis can be difficult, but the presence of fever and rash in addition to the characteristic eschar should be considered in the differential. This case also underscores the importance of maintaining a high index of suspicion for scrub typhus even in urban or atypical exposure settings, particularly in endemic regions.
